# Donepezil improves vascular function in a mouse model of Alzheimer’s disease

**DOI:** 10.1002/prp2.871

**Published:** 2021-10-29

**Authors:** Carolina Pellegrini, Vanessa D’Antongiovanni, Matteo Fornai, Emiliano Duranti, Filippo Baldacci, Nunzia Bernardini, Stefano Taddei, Agostino Virdis, Corrado Blandizzi, Stefano Masi, Luca Antonioli

**Affiliations:** ^1^ Department of Clinical and Experimental Medicine School of Medicine University of Pisa Pisa Italy; ^2^ Interdepartmental Research Center “Nutraceuticals and Food for Health” University of Pisa Pisa Italy

**Keywords:** Alzheimer's disease, endothelial function, mild cognitive impairment, oxidative stress

## Abstract

Cardiovascular complications in Alzheimer's disease (AD) patients can occur years to decades prior to the onset of clinical symptoms of the disease. Donepezil represents the most effective drug in the treatment of AD. However, the potential effect of donepezil on vascular function and structure remains largely unexplored. Here, we assessed the impact of donepezil on the vascular phenotype of an established model of accelerated senescence that develops spontaneously AD, the SAMP8 mouse. Three groups of animals were included: SAMR1 (control strain), SAMP8, and SAMP8 treated with donepezil. Treatment with donepezil was administered from the 4th to the 6th month of life. At 6 months, after cognitive tests by Morris Water Maze, animals were euthanized, and their mesenteric arteries were processed for functional experiments. Untreated SAMP8 developed cognitive impairment compared to SAMR1, while donepezil treatment significantly attenuated cognitive dysfunction. SAMP8 exhibited a higher media‐to‐lumen ratio than SAMR1 and donepezil‐treated animals. Endothelial function was impaired in SAMP8 animals compared to SAMR1. The addition of vitamin C improved the vasodilatory response to acetylcholine in SAMP8. Treatment with donepezil improved endothelial function in SAMP8 animals and reduced the additional vasodilation induced by vitamin C. In conclusion, in the SAMP8 AD model, cognitive impairment is associated with endothelial dysfunction and vascular remodeling which could contribute to cardiovascular events in AD since the prodromal phases of the disease. Treatment with donepezil alleviates vascular dysfunction associated with AD through an increase in NO availability likely by counteracting inflammation and oxidative stress.

**Abbreviations**
Ach: acetylcholine
Ach: acetylcholine;
AD: Alzheimer's disease
CNS: central nervous system
L‐NAME: Nω‐nitro‐ L‐arginine methylester
MWM: Morris water maze test
NA: noradrenaline
NO: nitric oxide
NOS: nitric oxide synthase
ROS: reactive oxygen species
SAMP8: senescence‐accelerated mouse prone 8
SAMR1: senescence‐accelerated mouse‐resistant 1

## 1 BACKGROUND

Dementia is a clinical syndrome characterized by a progressive decline in two or more cognitive domains, including memory, language, visuospatial function, personality, and behavior, which causes loss of abilities to perform instrumental and/or basic activities of daily living.^1^ Alzheimer's disease (AD) represents by far the most common cause of dementia and accounts for up to 80% of all dementia diagnoses.^1^

In attempting to ascertain the underlying etiology of AD, a commonly overlooked pathological aspect of the disease is the presence of concomitant cerebrovascular pathology, that might contribute to the process of neurodegeneration underpinning AD evolution.^2^ Indeed, AD patients show a marked cerebrovascular damage, characterized by brain small vessel disease that compromises blood flow distribution.^3^ These chronic vascular alterations seem to have an impact on amyloid clearance and neuronal metabolism.^2^ Several studies have described the presence of mitochondrial dysfunction, increased reactive oxygen species (ROS) production, and reduced nitric oxide (NO) bioavailability *via* ROS scavenging in the vessels of AD patients compared to controls.^4^

 At present, donepezil, an inhibitor of acetylcholinesterase, represent the most common drug used for the treatment of AD. Interestingly, beyond its capacity to increase acetylcholine (Ach) availability, donepezil might have anti‐inflammatory and free radical scavenging properties, thus suggesting a more complicated way of action of this drug.^5^

Based on these premises, it is conceivable that donepezil administration could potentially improve the vascular alterations commonly associated with AD and partially account for the cognitive benefits observed during treatment. However, the potential effect of donepezil on vascular function and structure remains largely unexplored. For this reason, this study was designed to examine the effect of donepezil administration on the vascular dysfunctions in a murine model of AD.

## 2 MATERIALS AND METHODS

### 2.1 Animals

SAMP8 mice (4 months old, 30–35 g body weight), a spontaneous genetic model of AD, and their control strain SAMR1 (4 months old, 30–35 g body weight) were purchased from ENVIGO Srl. The SAMP8 mouse is one of the accelerated senescence strains that develops spontaneously early memory and learning deficits, with similar features to those observed in AD patients.^6^

Animal care and handling were in accordance with the provisions of the European Community Council Directive 210/63/UE, recognized and adopted by the Italian Government. The experiments were approved by the Ethical Committee for Animal Experimentation of the University of Pisa and by the Italian Ministry of Health (authorization no. 865/2018‐PR).

### 2.2 Drug treatments

Based on the previous evidence^6^, ^7^ showing an impairment of cognitive and motor functions along with intestinal motor dysfunctions in SAMP8 mice starting from 6 months of age, we decided to focus the attention on SAMP8 animals at 6 months of age, in order to examine the effect of donepezil administration on the vascular dysfunctions since the early stages of AD. SAMP8 and SAMR1 animals at 4 months of age were treated orally with donepezil (3 mg/kg/day) for 2 months. SAMR1‐untreated animals (control group) and SAMP8‐untreated mice received only the drug vehicle.

The dose of donepezil was selected on the basis of a previous study.^8^ At the end of treatment, subgroups of animals were employed for Morris Water Maze (MWM) test in order to evaluate alterations of cognitive functions. One day after the end of cognitive tests, the animals were euthanized by cervical dislocation and mesenteric arteries were dissected and processed for functional experiments, as described below.

### 2.3 Evaluation of cognitive functions: MWM test

The MWM test was performed in accordance with the method previously described by Pellegrini et al.^6^ Briefly, mice were allowed to swim and find the visible platform within 60 s. Each animal was subjected to sessions of four trials every day for 2 days. Escape latency was collected for subsequent analysis. For the next 5 days, hidden‐platform training was performed by submerging the platform 1.5 cm below the surface of the water. Similarly, each animal was subjected to sessions of four trials every day. Finally, on the eighth day, the platform was removed from the tank for the probe trial. The number of target crossings and the entries into the target quadrant where the platform was placed were assessed in 60 s. Data were expressed as raw values.

### 2.4 Preparation of small mesenteric arteries and functional experiments

A second‐order branch of the mesenteric arterial tree was dissected and mounted on two‐glass microcannulae in a pressurized myograph (model 110P; Danish Myo Technology) as previously described.^9^ Media and lumen dimensions were measured with the intraluminal pressure maintained at 45 mmHg.

Endothelial‐dependent and independent vasorelaxation in all experimental groups were defined by the vasodilatory response to cumulative concentrations of Ach (0.001–100 μM). Vessels were precontracted with noradrenaline (NA, 10 μM). To ensure an equal NA‐induced contractility in vessels from SAMR1, SAMR1 treated with donepezil, SAMP8 and SAMP8 treated with donepezil mice, we performed preliminary experiments to assess the amount of vasoconstriction induced by increasing NA concentrations (from 1 to 100 μM). To evaluate the contribution of NO availability and ROS production to the endothelial function, ACh concentration–response curves were constructed before and after 30‐min preincubation with the NOS inhibitor Nω‐nitro‐L‐arginine methyl ester (L‐NAME, 100 μM; Sigma‐Aldrich) or the antioxidant ascorbic acid (100 μM, 30‐min preincubation; SigmaAldrich). Vessels were then deactivated by perfusion with Ca^2+^‐free PSS containing 10 mmol/L EGTA for 30 min. Media thickness and lumen diameter were measured in three different points from each small artery to obtain the media‐lumen ratio (M/L), with a constant intraluminal pressure of 45 mmHg (Virdis et al., 2003). This parameter is an index of structural abnormalities in resistance arteries. An increment of M/L ratio is associated with an inward eutrophic remodeling of the vessel wall resulting in an increased risk of developing cardiovascular complications. Media cross‐sectional area (MCSA), an index of hypertrophic remodeling of vascular wall, was obtained by subtraction of the internal from the external cross‐sectional areas using external plus lumen diameters, as previously described (Bruno et al., 2017).

To confirm a potential influence of donepezil on vascular function and its determinants, vessels from SAMP8 (*n* = 3) and from SAMR1 (*n* = 3) mice were acutely incubated with donepezil alone or in combination with ascorbic acid and L‐NAME for 30 min. ACh concentration–response curves were subsequently repeated.

### 2.5 Statistical analysis

Data are presented as mean ± SEM and analyzed by GraphPad Prism 7.0 (GraphPad Software Inc.). Statistical significances were determined by two‐way analysis of variance followed by post hoc analysis with the Fisher LSD test or one‐way ANOVA followed by Tukey's post hoc test where appropriate. A *P* value <.05 was considered significantly different.

## 3 RESULTS

### 3.1 Evaluation of cognitive functions (MWM test)

The administration of donepezil to SAMR1 mice did not elicit any significant change in cognitive functions (data not shown). Therefore, we adopted the animals treated with donepezil vehicle mice as a control group for the evaluation of the drug under investigation, designed as a test drug.

**FIGURE 1:**
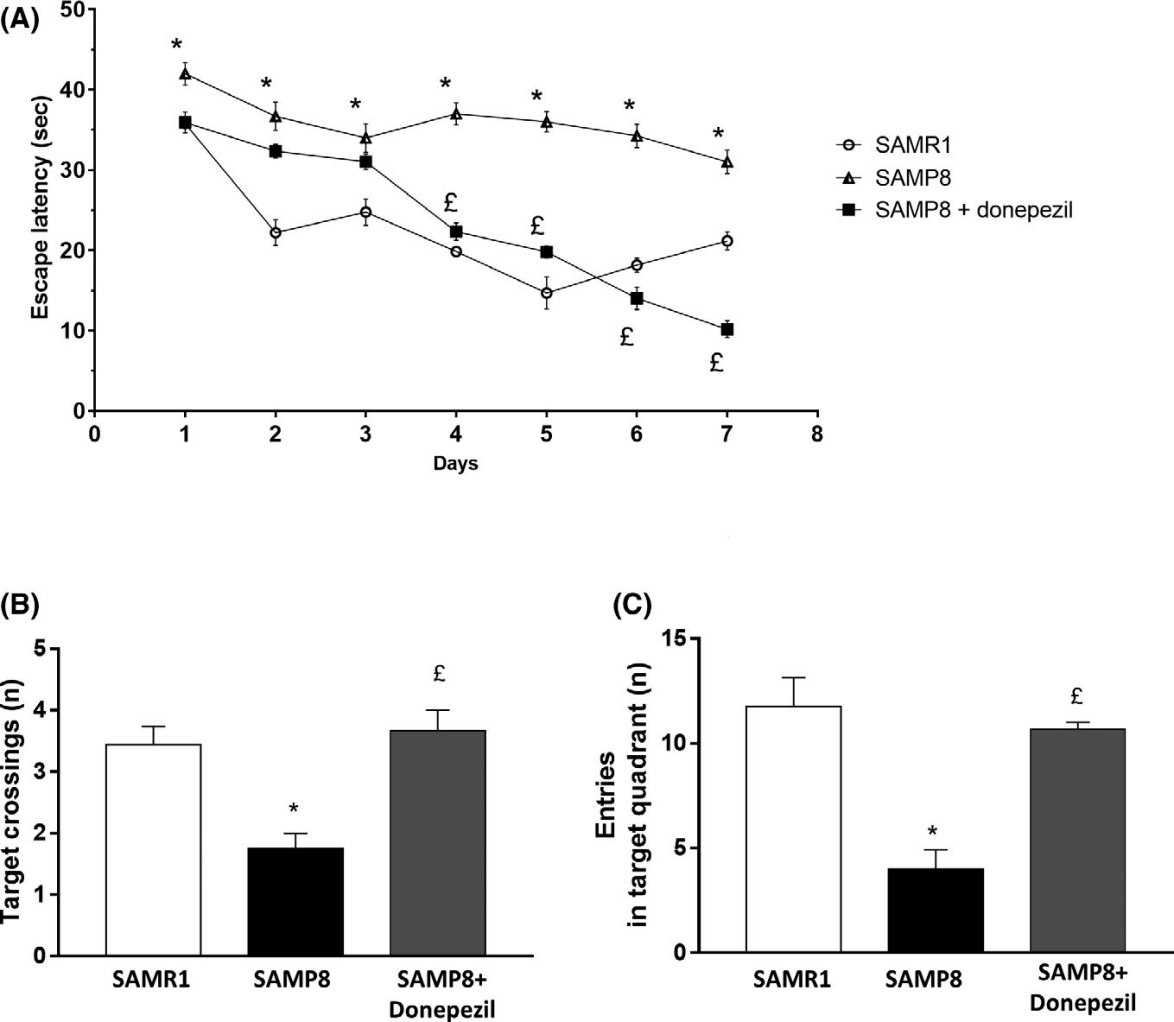
Cognitive performance of SAMR1 (*n* = 5), SAMP8 (*n* = 5), and SAMP8 treated with donepezil (*n* = 4), during the training and probe trial session of the Morris water maze test. (A) Escape latency in SAMR1, SAMP8 and SAMP8 treated with donepezil mice, during seven consecutive days of Morris water maze test training; (B) number of target crossings and (C) entries into the target quadrant. Data are expressed as mean ± SEM obtained from six animals. Differences among groups were evaluated by two‐way analysis of variance followed by post hoc analysis with the Fisher LSD test or a one‐way ANOVA followed by Tukey's post hoc test where appropriate. *p < .05, significant differences versus age‐matched SAMR1; ^£^ *p* < .05, significant differences versus age‐matched SAMP8

During the training test, SAMP8 mice at 6 months of age displayed a significant increase in the escape latency time, as compared with SAMR1 mice (Figure 1A). By contrast, no significant differences were observed among the training days in SAMR1 mice (Figure 1A). Treatment of SAMP8 mice with donepezil was associated with a significant decrease in the escape latency time (Figure 1A).

During the probe trial, the number of target crossings as well as the entries into the target quadrant were significantly decreased in SAMP8 mice, as compared with SAMR1 mice (Figure 1B,C). Treatment of SAMP8 mice with donepezil was associated with a significant increase in the number of target crossings as well as the entries into the target quadrant, as compared with SAMP8‐untreated mice (Figure 1B,C).

### 3.2 Endothelium‐dependent vascular relaxation and remodeling

**FIGURE 2:**
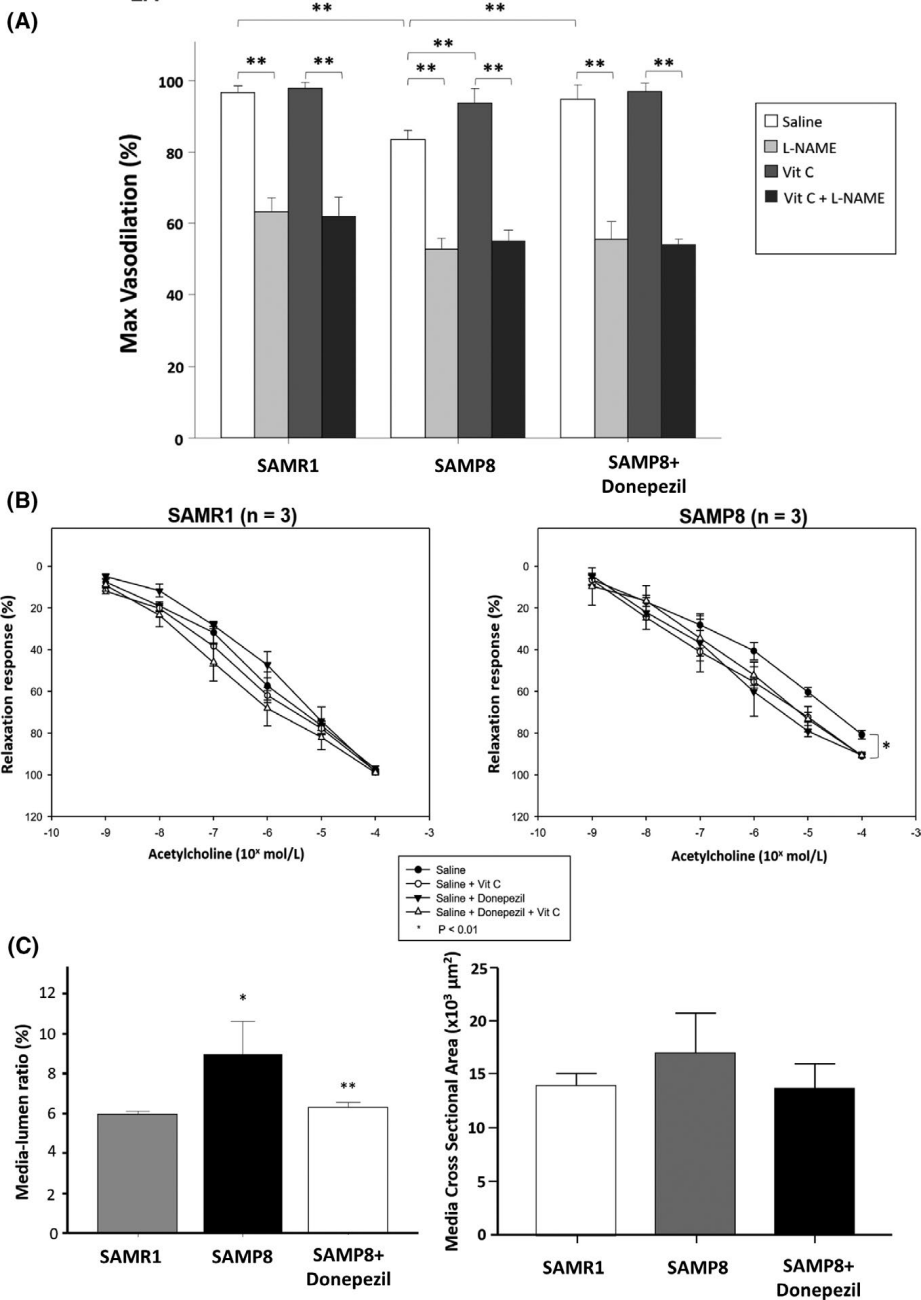
Vascular results. (A) Endothelial‐dependent relaxations of mesenteric resistance arteries to acetylcholine alone, or in combination with ascorbic acid or L‐NAME in SAMR1 (*n* = 5), SAMP8 (*n* = 5), and SAMP8 chronically treated with donepezil (*n* = 4) mice. Data are expressed as mean ± SD. Differences among groups were evaluated by one‐way ANOVA followed by Tukey's post hoc test **p < .001. (B) Effects on endothelial function of acute incubation with donepezil with or without ascorbic acid in vessels from SAMR1 (*n* = 3) or SAMP8 untreated (*n* = 3) mice. Data are expressed as mean ± SD. Differences among groups were evaluated by one‐way ANOVA followed by Tukey's post hoc test *p < .05. (C) Differences of Median‐Lumen (M/L) ratio and Media Cross Sectional Area (MCSA) between SAMR1 (*n* = 5), SAMP8 (*n* = 5) and SAMP8 supplemented with donepezil (*n* = 4) mice. Data are expressed as mean ± SD. Differences among groups were evaluated by one‐way ANOVA followed by Tukey's post hoc test. *p < .001 versus SAMR1; **p < .001 versus SAMP8

In mesenteric vessels from SAMR1, relaxation to ACh was significantly attenuated by L‐NAME (100 µM), and not affected by ascorbic acid (100 µM) (Figure 2A). Treatment of SAMR1 mice with donepezil did not modify the patterns of responses to ACh in mesenteric vessels, in the absence or in the presence of ascorbic acid (data not shown). Mesenteric vessels from SAMP8 mice showed a reduced vasorelaxation to ACh as compared to SAMR1 animals, and also the inhibitory effect of L‐NAME on ACh‐induced relaxation was significantly attenuated as compared to SAMR1 mice (Figure 2A). ACh‐dependent relaxation in SAMP8 mice was improved by pre‐incubation with ascorbic acid (Figure 2A). Chronic supplementation with donepezil preserved the endothelial function of SAMP8 mice, leading to levels of Ach‐dependent vasodilation similar to those observed in SAMR1 mice (Figure 2A). Incubation with L‐NAME blunted the positive effects of donepezil chronic supplementation on endothelial function of SAMP8 mice, to a similar extent than that observed in SAMR1 mice. Furthermore, the addition of ascorbic acid on vessels of SAMP8 mice underwent chronic supplementation with donepezil did not cause further vasodilation (Figure 2A).

In the experiments testing the capacity of donepezil to induce an acute vasodilatory responses, SAMP8 mice showed again a reduced vasodilatory response to Ach than SAMR1 mice (Figure 2B), confirming the presence of endothelial dysfunction. The acute incubation of vessels from SAMR1 mice with donepezil did not affect the response to Ach, while in SAMP8 mice it caused a significant improvement in the Ach‐dependent vasodilation (Figure 2B). The addition of ascorbic acid to vessels of SAMP8 mice pretreated with donepezil did not cause a significant improvement of the Ach‐dependent vasodilation as it was observed, instead, in untreated vessels from the same mice (Figure 2B).

SAMP8 mice showed an increase in the M/L ratio, as compared with SAMR1 mice (Figure 2C). Treatment of SAMP8 with donepezil prevented the increase in the M/L ratio, leading this parameter to levels similar to those observed in SAMR1 mice (Figure 2C). No significant differences were observed in MCSA among the different groups (Figure 2C).

## 4 DISCUSSION

To extend current knowledge about the effects of acetylcholinesterase inhibitors in counteracting vascular remodeling and endothelial dysfunction associated with AD, in this study, we examined the effects of donepezil on the vascular phenotype of an established model of AD. In particular, the SAMP8 mouse, which develops spontaneously early learning and memory deficits starting from 6 months of age with similar features to AD patients, can be an extremely valuable model to investigate cardiovascular symptoms in the prodromal phases of AD.^6^, ^7^

In the first set of experiments, we demonstrated that mesenteric small arteries from SAMP8 animals showed an increased M/L ratio as compared with SAMR1 animals. In addition, SAMP8 mice were characterized by altered endothelium‐dependent relaxations likely depending on a decrease in NO availability. Such NO reduction could be associated with an increase in intravascular ROS levels, as documented by the restored response to ACh after pre‐incubation with ascorbic acid. According to the current results, it has been observed that endothelial dysfunction in the aorta from SAMP8 mice was associated with a decrease in eNOS expression and NO production.^10^

Based on these findings, it is conceivable the occurrence of vascular oxidative stress in the prodromal phase of AD could contribute to a generalized endotheliopathy which is detectable also at the level of the central nervous system. Indeed, clinical evidence reported a correlation among AD, increased systemic oxidative stress and inflammation and compromised vascular function.^5^

Of note, a greater endothelial dysfunction in human small vessels is associated with a more severe microvascular remodeling.^11^ Therefore, the improved endothelial function observed in SAMP8 mice treated with donepezil could explain the reduced M/L observed in the same animals as compared to the untreated group. This hypothesis is supported by recent studies showing that donepezil, besides inhibiting acetylcholinesterase, attenuates the vascular dysfunction associated with AD, by counteracting oxidative stress and inflammation.^5^, ^12^

Based on the above considerations, in the second part of this study, we focused our attention on characterizing the potential mechanisms through which donepezil could counteract the endothelial dysfunction in SAMP8 animals. In particular, the improvement of endothelial function observed in mesenteric vessels from SAMP8 animals treated with donepezil was abolished by incubation of the vessel with L‐NAME, while ascorbic acid was no longer able to affect the vascular response to Ach. These results suggest the beneficial effects of donepezil on endothelial functions could be ascribed to its ability to increase NO availability, probably through a reduction of the vascular levels of oxidative stress. To confirm this hypothesis, we show that the acute in vitro incubation of mesenteric arteries with donepezil improved endothelial function, and, similarly to what was observed with the chronic supplementation, this response was abrogated by L‐NAME and was not affected by ascorbic acid incubation. The separation between Ach‐curves in arteries from SAMP8 mice incubated with and without donepezil occurred from Ach concentrations in the order of 10^−7^ mmol/L (the third point of the dose‐response curves). The limited capacity to identify clear changes in the vasorelaxation at lower doses of Ach might depend on the limited ability of the myographic technique to capture minimal changes in the diameter of the arteries, combined with the limited number of animals included in the experiments. However, these results could also depend on a potential limited benefits of the antioxidant capacity of donepezil on the endothelial‐dependent vasodilation, requiring supra‐physiological doses of Ach to become clearly evident. While this is a possibility, the chronic supplementation experiments documented that the magnitude of endothelial function improvement induced by donepezil is probably sufficient to induce long term positive changes on the vascular remodeling associated with AD, and that this is likely due to its anti‐oxidant and anti‐inflammatory properties, resulting in a preserved NO availability within the vascular wall. The exact molecular mechanisms through which donepezil preserve NO availability remains to be elucidated and requires further investigation. In addition, the potential benefits of therapies that improve endothelial function on the cognitive decline related to AD requires confirmation by means of additional experimental approaches.

In conclusion, this study provides evidence that, in the SAMP8 AD model, cognitive impairment is associated with endothelial dysfunction and vascular remodeling which could contribute to cardiovascular events in AD patients since the prodromal phases of the disease. In this context, donepezil alleviates vascular dysfunction associated with AD through an increase in NO availability likely by counteracting inflammation and oxidative stress.

Overall, these findings set the ground for better understanding the mechanisms underlying vascular dysfunction in AD and pave the way to the identification of novel pharmacological approaches for the management of cardiovascular disorders associated with AD.

## DISCLOSURE

The authors declare that they have no conflict of interest.

## AUTHORS CONTRIBUTIONS

CP, LA, MF, and MS conceived the study; ED, VD, and CP carried out the experiments, FB, SM, and ED collected and analyzed data; CP and VD, wrote the manuscript with input from all other authors. NB, ST, AV, and CB revised the manuscript. All authors approved the manuscript.

## ETHICS APPROVAL

All experiments were conducted under the institutional guidelines. Animal care and handling were in accordance with the provisions of the European Community Council Directive 210/63/UE, recognized and adopted by the Italian Government. The experiments were approved by the Ethical Committee for Animal Experimentation of the University of Pisa and by the Italian Ministry of Health (authorization no. authorization no. 865/2018‐PR).

## Data Availability

All raw data used and analyzed for this study are available from the corresponding author on request.
